# Characterization and Valuation of the Uncertainty of Calibrated Parameters in Microsimulation Decision Models

**DOI:** 10.3389/fphys.2022.780917

**Published:** 2022-05-09

**Authors:** Fernando Alarid-Escudero, Amy B. Knudsen, Jonathan Ozik, Nicholson Collier, Karen M. Kuntz

**Affiliations:** ^1^ Division of Public Administration, Center for Research and Teaching in Economics (CIDE), Aguascalientes, Mexico; ^2^ Institute for Technology Assessment, Massachusetts General Hospital, Boston, MA, United States; ^3^ Decision and Infrastructure Sciences Division, Argonne National Laboratory, Argonne, IL, United States; ^4^ Consortium for Advanced Science and Engineering, University of Chicago, Chicago, IL, United States; ^5^ Division of Health Policy and Management, University of Minnesota School of Public Health, Minneapolis, MN, United States

**Keywords:** microsimulation models, uncertainty quantification, calibration, Bayesian, value of information analysis, decision-analytic models, high-performance computing, EMEWS

## Abstract

**Background:** We evaluated the implications of different approaches to characterize the uncertainty of calibrated parameters of microsimulation decision models (DMs) and quantified the value of such uncertainty in decision making.

**Methods:** We calibrated the natural history model of CRC to simulated epidemiological data with different degrees of uncertainty and obtained the joint posterior distribution of the parameters using a Bayesian approach. We conducted a probabilistic sensitivity analysis (PSA) on all the model parameters with different characterizations of the uncertainty of the calibrated parameters. We estimated the value of uncertainty of the various characterizations with a value of information analysis. We conducted all analyses using high-performance computing resources running the Extreme-scale Model Exploration with Swift (EMEWS) framework.

**Results:** The posterior distribution had a high correlation among some parameters. The parameters of the Weibull hazard function for the age of onset of adenomas had the highest posterior correlation of −0.958. When comparing full posterior distributions and the maximum-a-posteriori estimate of the calibrated parameters, there is little difference in the spread of the distribution of the CEA outcomes with a similar expected value of perfect information (EVPI) of $653 and $685, respectively, at a willingness-to-pay (WTP) threshold of $66,000 per quality-adjusted life year (QALY). Ignoring correlation on the calibrated parameters’ posterior distribution produced the broadest distribution of CEA outcomes and the highest EVPI of $809 at the same WTP threshold.

**Conclusion:** Different characterizations of the uncertainty of calibrated parameters affect the expected value of eliminating parametric uncertainty on the CEA. Ignoring inherent correlation among calibrated parameters on a PSA overestimates the value of uncertainty.

## Background

Decision models (DMs) are commonly used in cost-effectiveness analysis where uncertainty in the parameters is inherent (Kuntz et al., 2017). The impact of parameter uncertainty can be assessed with a probabilistic sensitivity analysis (PSA) to characterize decision uncertainty (i.e., the probability of a strategy being cost-effective) (Briggs et al., 2012; Sculpher et al., 2017) and to quantify the value of potential future research by determining the potential consequences of a decision with value of information (VOI) analysis (Schlaifer, 1959; Raiffa and Schlaifer, 1961).

The parameters of DMs can be split into two categories, those obtained from the literature or estimated from available data (i.e., external parameters) and those that need to be estimated through calibration (i.e., calibrated parameters). External parameters are estimated either from individual-level or aggregated data that directly inform the parameters of interest. There are recommendations on the type of distributions that characterize their uncertainty based on the characteristics of the parameters or the statistical model used to estimate them (Briggs et al., 2012). For example, a probability could be modeled with a beta distribution and a relative risk with a lognormal distribution (Briggs et al., 2002). For calibrated parameters, no such data exist that can directly inform their uncertainty because a research study hasn’t been conducted or is unfeasible to conduct, or because the parameters reflect unobservable phenomena, as is often the case in natural history models of chronic diseases (Welton and Ades, 2005; Karnon et al., 2007; Rutter et al., 2009; Rutter et al., 2011) or in infectious disease dynamic models (Enns et al., 2017). The choice of distribution for these parameters is often less clear. One option is to define uniform distributions with wide bounds or generate informed distributions based on moments of the calibrated parameters, such as the mean and standard error. However, the impact of these approaches to characterize the uncertainty of calibrated parameters on decision uncertainty and the VOI on reducing that uncertainty has not been studied.

Model calibration is the process of estimating unobserved or unobservable parameters by matching model outputs to observed clinical or epidemiological data (known as calibration targets) (Kennedy and O’Hagan, 2001; Stout et al., 2009; Kuntz et al., 2017). While there are several approaches for searching the parameter space in the calibration process, most approaches are insufficient to characterize the uncertainty in the calibrated model parameters because they do not provide interval estimates. For example, direct-search optimization algorithms like Newton-Raphson Nelder-Mead (Nelder and Mead 1965) simulated annealing or genetic algorithms (Kong et al., 2009) treat the calibration targets as if they were known with certainty, so are primarily useful when identifying a single or a set of parameters that yield good fit to the targets (Kennedy and O’Hagan, 2001).

A sample of calibrated parameter sets that correctly characterizes the uncertainty of the calibration target data is obtained from their joint distribution, conditional on the calibrated targets. To obtain the joint distribution, calibration could be specified as a statistical estimation problem under at least two different frameworks, through maximum likelihood (ML) or Bayesian methods. ML can fail in obtaining interval estimates by not being able to estimate the Hessian matrix when the likelihood is intractable or computationally intensive to simulate and when the calibration problem is non-identifiable (Gustafson, 2005; Alarid-Escudero et al., 2018); thus, we focus on Bayesian methods (Romanowicz et al., 1994; Kennedy and O’Hagan, 2001; Oakley and O’Hagan, 2004; Gustafson, 2005; Kaipio and Somersalo, 2005; Oden et al., 2010; Gustafson, 2015; Alarid-Escudero et al., 2018).

Despite their suitability to correctly characterize the uncertainty of calibrated model parameters, Bayesian methods are generally computationally expensive because they require evaluating the model thousands and sometimes millions of times. The computational burden of Bayesian methods does not seem to be an impediment when calibrating non-computationally intensive DMs (e.g., Markov cohort models, difference equations, relatively small systems of differential equations, etc.) (Whyte et al., 2011; Hawkins-Daarud et al., 2013; Jackson et al., 2016; Menzies et al., 2017). Still, they become more challenging to apply to DMs that could be computationally intensive to solve, such as models that simulate underlying stochastic processes (Iskandar, 2018) (e.g., microsimulation, discrete-event simulation, and agent-based models), limiting their use to only a few of such models (Rutter et al., 2009).

However, the increasing availability of high-performance computing (HPC) systems in an academic, national laboratory and commercial settings enables such systems for model calibration and model exploration of microsimulation DMs at a large scale to a broader audience. HPC resources allow running large numbers of DMs concurrently, allowing calibration algorithms to generate large batches of parameters simultaneously, such as the incremental mixture importance sampling (IMIS) described below, to be run efficiently. In many cases, particularly in the academic and national laboratory settings, computing allocations can be obtained through proposals with no cost to researchers (e.g., the Advanced Scientific Computing Research (ASCR) Leadership Computing Challenge (ALCC), https://science.osti.gov/ascr/Facilities/Accessing-ASCR-Facilities/ALCC). However, implementing dynamic calibration algorithms for HPC resources has generally proved difficult, requiring specialized knowledge across various disciplines. The Extreme-scale Model Exploration with Swift (EMEWS) framework was designed to facilitate large-scale model calibration and exploration on HPC resources (Ozik et al., 2016a) to a broad community. EMEWS can run very large, highly concurrent ensembles of microsimulation DMs of varying types with a broad class of calibration algorithms, including those increasingly available to the community via. Python and R libraries, using HPC workflows. EMEWS workflows provide interfaces for plugging in DMs (and any other simulation or black box model) and algorithms, through an inversion of control scheme (Ozik et al., 2018), to control the dynamic execution of those DMs for calibration and other heuristics for “model exploration” purposes. These interfaces help reduce the need for an in-depth understanding of how task coordination and inter-task dependencies are implemented for HPC resources. The general use of EMEWS can be seen on the EMEWS website (https://emews.github.io), which includes links to tutorials.

The purpose of our study is threefold. First, to use recently developed HPC capabilities to characterize the uncertainty of calibrated parameters of a microsimulation model of the natural history of colorectal cancer (CRC). Second, to explore the impact of different approaches to characterize the uncertainty of calibrated parameters on decision uncertainty, and third, to use VOI analysis to quantify the value of eliminating parameter uncertainty when assessing the cost-effectiveness of CRC screening.

## Methods

We developed a microsimulation model of the natural history of CRC and calibrated it using a Bayesian approach. We then overlaid a simple CRC screening strategy onto the natural history model and conducted a cost-effectiveness analysis (CEA) of screening, including a PSA. Instead of using the posterior means to represent the best estimates of each calibrated parameter, we obtained the posterior distribution using a Bayesian approach that represents the joint uncertainty of all the calibrated parameters that can then be used in a PSA. We then evaluated the impact of different approaches to characterize the uncertainty of calibrated parameters on the joint distribution of incremental costs and incremental effects of the screening strategy compared with no screening through a PSA while also accounting for the uncertainty of the external parameters (e.g., test characteristics, costs, etc.). Finally, we quantified the amount of money that a decision maker should be willing to spend to eliminate all parameter uncertainty (i.e., the expected value of perfect information (EVPI)).

### Microsimulation Model of the Natural History of CRC

**FIGURE 1:**
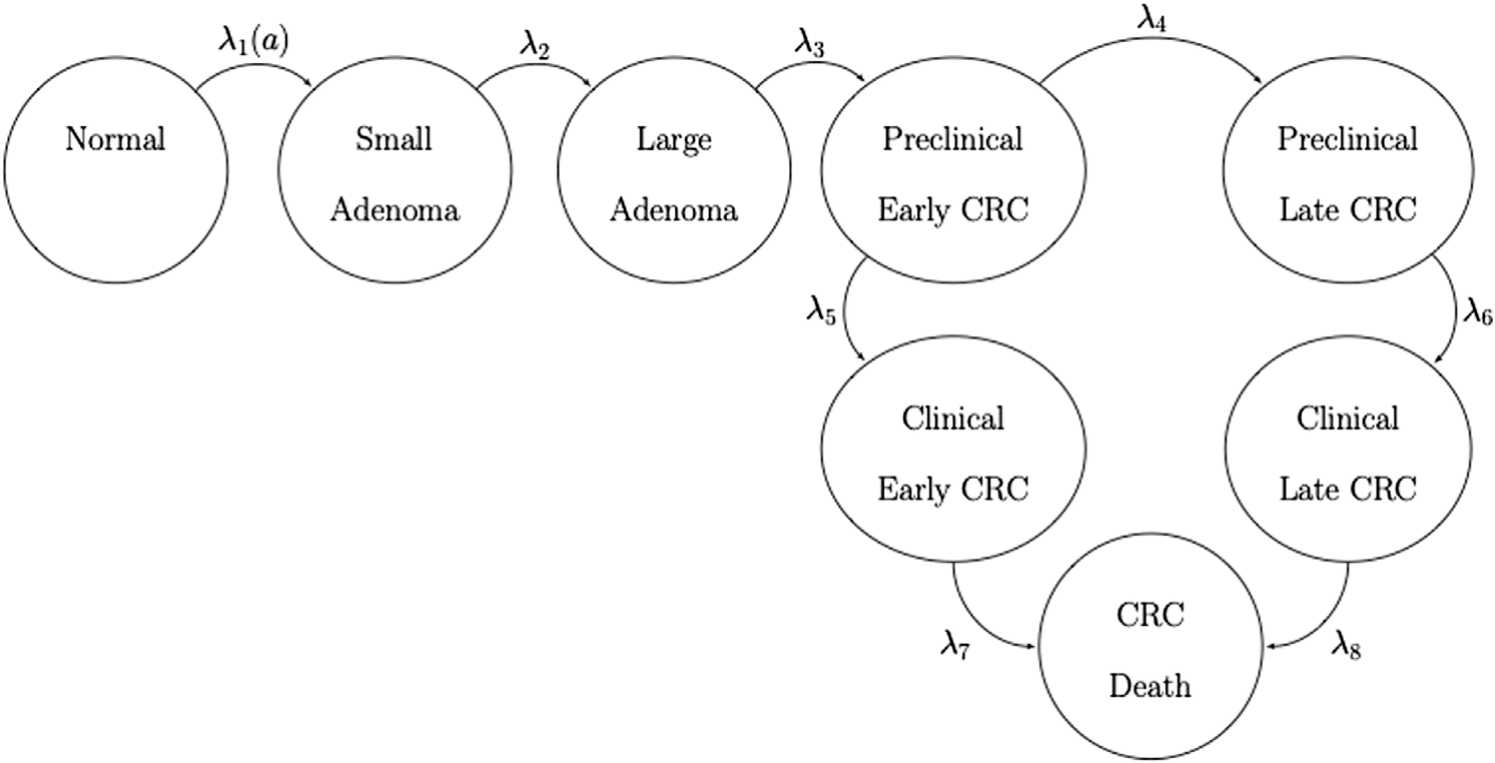
State-transition diagram of the nine-state microsimulation model of the natural history of colorectal cancer. Individuals in all health states face an age-specific mortality of dying from other causes (state not shown) (Jalal et al., 2021).

We developed a state-transition microsimulation model of the natural history of CRC implemented in R (Krijkamp et al., 2018) based on a previously developed model (Alarid-Escudero et al., 2018). The progression between health states follows a continuous-time age-dependent Markov process. There are two age-dependent transition intensities (i.e., transition rates), $\lambda_{1}\left(a\right)$ and μ(a) , that govern the age of onset of adenomas and non-cancer-specific mortality, respectively. Following Wu et al. (2006) we specify $\lambda_{1}\left(a\right)$ as a Weibull hazard with the following specification

$$\lambda_{1}\left(a\right)=l\gamma a^{\gamma -1},$$

where a is the age of the simulated individuals, and l and γ are the scale and shape parameters of the Weibull hazard function, respectively. The model simulates two adenoma categories: small (adenoma smaller than 1 cm in size) and large (adenoma larger than or equal to 1 cm in size). All adenomas start small and can transition to the large size category at a constant annual rate $\lambda_{2}$ . Large adenomas may become preclinical CRC at a constant annual rate $\lambda_{3}$ . Both small and large adenomas may progress to preclinical CRC, although most will not in a simulated individual’s lifetime. Early preclinical cancers (preclinical stages I and II) progress to late stages (preclinical stages III and IV) at a constant annual rate $\lambda_{4}$ and could become symptomatic at a constant annual rate $\lambda_{5}$ . Late preclinical cancer could become symptomatic at a constant annual rate $\lambda_{6}$ . After clinical detection, the model simulates the survival time to early and late CRC death using cancer-specific constant mortality rates, $\lambda_{7}$ and $\lambda_{8}$ , respectively. The model has nine health states: normal, small adenoma, large adenoma, early preclinical CRC, late preclinical CRC, early clinical CRC, late clinical CRC, CRC death, and death from other causes. The state-transition diagram of the continuous-time model is shown in Figure 1.

The continuous-time age-dependent Markov process of this natural history model of CRC can be represented by an age-dependent 9×9 transition intensity matrix, Q(a) . To translate Q(a) to discrete-time, we compute the annual-cycle age-dependent transition probability matrix, P(a,t) , using the Kolmogorov differential equations (Kolmogorov, 1963; Cox and Miller, 1965; Welton and Ades, 2005)

P(a,t)=Exp(tQ(a)),

where t=1 and Exp() is the matrix exponential. In discrete time, the natural history model of CRC allows individual transitions across multiple health states in a single year. Small and large adenomas may progress to preclinical or clinical CRC, and preclinical cancers may progress through early and late stages.

We simulated a hypothetical cohort of 50-year-old women in the United States over a lifetime. The cohort starts the simulation with a prevalence of adenoma of $p_{adeno}$ , from which a proportion, $p_{small}$ , corresponds to small adenomas, and a prevalence of preclinical early and late CRC of 0.12% (Rutter et al., 2007) and 0.08% (Wu et al., 2006), respectively. The parameters $p_{adeno}$ and $p_{small}$ are calibrated parameters. The simulated cohort is at risk of all-cause mortality, μ(a) , from all health states obtained from 2014 United States life tables (Arias et al., 2017).

### Calibration Targets

We used the microsimulation model of the natural history of CRC to generate synthetic calibration targets by selecting a set of parameter values based on plausible estimates from the literature (Table 1) (Wu et al., 2006; Rutter et al., 2007). We simulated four different age-specific synthetic targets, including adenoma prevalence, the proportion of small adenomas, and CRC incidence for early and late stages, which resemble commonly used calibration targets for this type of model (Rutter et al., 2009; Whyte et al., 2011; Frazier et al., 2000; Kuntz et al., 2011). To simulate the calibration targets, we ran the microsimulation model 100 times to get a stable estimate of the standard errors (SEs) using the fixed values in Table 1. We then aggregated each outcome across all 100 model replications to compute their mean and SE. To account for different levels of uncertainty across targets given the amount of data to estimate their summary measures, we simulated various targets based on cohorts of different sizes (Rutter et al., 2009). Adenoma-related targets were based on a cohort of 500 individuals, and cancer incidence targets were based on 100,000 individuals.

**TABLE 1:** Description of parameters of the natural history model.

Symbol	Description	Value	Source	Prior distribution	Calibrated
Initial state of 50-year-old cohort					
Proportions					
$p_{adeno}$	Prevalence of adenoma at age 50	0.25	Rutter et al. (2007)	Beta(3, 8)	Yes
$p_{small}$	Proportion adenomas that are small at age 50	0.71	Wu et al. (2006)	Beta(6, 3)	Yes
—	Prevalence of preclinical early CRC at age 50	0.12	Wu et al. (2006)	Fixed	No
—	Prevalence of preclinical late CRC at age 50	0.08	Wu et al. (2006)	Fixed	No
Disease dynamics					
Transition rates (annual)					
l	Scale parameter of Weibull hazard	2.86e-06	Wu et al. (2006)	Log-normal(m = −11.97, s = 0.59)	Yes
γ	Shape parameter of Weibull hazard	2.78	Wu et al. (2006)	Log-normal(m = 1.04, s = 0.18)	Yes
$\lambda_{2}$	Small adenoma to large adenoma	0.0346	Wu et al. (2006)	Log-normal(m = −3.45, s = 0.59)	Yes
$\lambda_{3}$	Large adenoma to preclinical early CRC	0.0215	Wu et al. (2006)	Log-normal(m = −3.91, s = 0.35)	Yes
$\lambda_{4}$	Preclinical early CRC to preclinical late CRC	0.3697	Wu et al. (2006)	Log-normal(m = −1.15, s = 0.23)	Yes
$\lambda_{5}$	Preclinical early CRC to clinical early CRC	0.2382	Wu et al. (2006)	Log-normal(m = −1.41, s = 0.10)	Yes
$\lambda_{6}$	Preclinical late CRC to clinical late CRC	0.4582	Wu et al. (2006)	Log-normal(m = −0.78, s = 0.22)	Yes
$\lambda_{7}$	CRC mortality in early stage	0.0302	Wu et al. (2006)	Fixed	No
$\lambda_{8}$	CRC mortality in late stage	0.2099	Wu et al. (2006)	Fixed	No
μ(a)	Age-specific mortality	Age-specific	Arias, (2017)	Fixed	No

### Calibration of the Microsimulation Model of the Natural History

To state the calibration of the microsimulation model as an estimation problem (Alarid-Escudero et al., 2018), we define M as the microsimulation model of the natural history of CRC with 11 input parameters. Cancer-specific mortality rates from early and late stages of CRC could be obtained from cancer population registries (e.g., the Surveillance, Epidemiology and End Results (SEER) registry in the United States), so calibration of these rates was unnecessary. That is, $\theta_{k}=\left[\lambda_{7},\lambda_{8}\right]$ is a set of 2 parameters that are either known or could be obtained from external data (i.e., are external parameters). The model has a set of 9 parameters $\theta_{u}=\left[p_{adeno},p_{small},l,\gamma ,\lambda_{2},\lambda_{3},\lambda_{4},\lambda_{5},\lambda_{6}\right]$ that cannot be directly estimated from sample data and need to be calibrated. M 's full set of parameters is $\theta =\left[\theta_{u},\theta_{k}\right]$ .

To calibrate M , we adopted a Bayesian approach that allowed us to obtain a joint posterior distribution that characterizes the uncertainty of both the calibration targets and previous knowledge of the parameters of interest in the form of prior distributions. Prior distributions can reflect experts’ opinions, or when little knowledge is available, these could be specified as uniform distributions. We constructed the likelihood function by assuming that each type of target t , including adenoma prevalence, proportion of small adenomas, early clinical CRC incidence, and late clinical CRC incidence for each age group a , $y_{t_{a}}$ , are normally distributed with mean $\phi_{t_{a}}$ and standard deviation $\sigma_{t_{a}}$ (Alarid-Escudero et al., 2018). That is,

$$y_{t_{a}}∼Normal\left(\phi_{t_{a}},\sigma_{t_{a}}\right),$$

where $\phi_{t_{a}}\mathrm{=E}\left[M\left(\theta \right)\right]$ is the expected value of the model-predicted output from parameter set θ . We added the log-likelihoods across all targets to compute an aggregated likelihood measure. We defined prior distributions for all $\theta_{u}$ based on previous knowledge or the nature of the parameters (Table 1). We defined beta distributions for the prevalence of adenomas and the proportion of small adenomas at age 50, bounded between 0 and 1. We assumed that the annual transition rates follow a log-normal distribution for their priors, defined over positive numbers. The ranges given in Table 1 are assumed to represent the 95% equal-tailed interval for the beta and log-normal distributions.

To conduct the Bayesian calibration, we used the incremental mixture importance sampling (IMIS) algorithm (Steele et al., 2006; Raftery and Bao, 2009), which has been previously used to calibrate health policy models (Menzies et al., 2017; Ryckman et al., 2020). We ran the IMIS algorithm on the Midway2 cluster at the University of Chicago Research Computing Center (https://rcc.uchicago.edu/resources/high-performance-computing). Midway2 is a hybrid cluster, including both central processing unit (CPU) and graphics processing unit (GPU) resources. For this work, we used the CPU resources. Midway2 consists of 370 nodes of Intel E5-2680v4 processors, each with 28 cores and 64 GB of RAM. Using EMEWS, we developed a workflow that parallelized the likelihood evaluations over 1,008 processes using 36 compute nodes. In other words, we reduced the computation time approximately by 250 had the analysis been conducted in a laptop with four processing cores.

Consistent with previous analyses, we deemed that convergence had occurred when the target effective sample size (ESS) got as close as 5,000 (Rutter et al., 2019; DeYoreo et al., 2022). An advantage of IMIS over other Monte Carlo methods, such as Markov chain Monte Carlo, is that with IMIS, we parallelize the evaluation of the likelihood for different sampled parameter sets, making its implementation perfectly suitable for an HPC environment using EMEWS. IMIS requires defining and computing the likelihood, which we could do with our model. However, when computing the likelihood is intractable, modelers could use the incremental mixture approximate Bayesian computation (IMABC) algorithm (Rutter et al., 2019), which an approximate Bayesian version of IMIS.

### Propagation of Uncertainty

We sampled 5,000 parameter sets from the IMIS joint posterior distribution for the nine calibrated model parameters. To compare the outputs of the calibrated model against the calibration targets, we propagated the uncertainty of the calibrated parameters through the microsimulation model of the natural history of CRC. We simulated a cohort of 100,000 (i.e., the largest cohort size used to generate the targets). We generated the model-predicted adenoma and cancer outcomes for each of the 5,000 calibrated parameter sets drawn from their joint posterior distribution. We computed the 95% posterior predicted interval (PI), defined as the estimated range between the 2.5th and 97.5th percentiles of the model-predicted posterior outputs to quantify the uncertainty limit model outputs.

### Cost-Effectiveness Analysis of Screening for CRC

With the calibrated microsimulation model of the natural history of CRC, we assessed the cost-effectiveness of 10-yearly colonoscopy screening starting at age 50 years compared to no screening. For adenomas detected with colonoscopy, a polypectomy was performed during the procedure. Individuals diagnosed with a small or large adenoma underwent surveillance with colonoscopy every 5 or 3 years, respectively. We assumed screening or surveillance continued until 85 years of age. Individuals with a history of polyp diagnosis had higher recurrence rates after polypectomy, that is, a higher transition rate from normal to small adenoma (i.e., $\lambda_{1}\left(a\right)$ ). We assumed a hazard ratio of 2 for small adenomas and 3 for the large adenomas. The costs and utilities of CRC care varied by stage, and individuals without clinical CRC had a utility of 1. Table 2 shows the parameters used in the CEA with their corresponding distributions.

**TABLE 2:** Description of cost-effectiveness analysis parameters.

Parameter	Value (range)	Distribution	Source
Screening test characteristics (location-specific)			
Small adenomas			
Sensitivity	0.773 (0.734–0.808)	Beta	Van Rijn et al. (2006)
Specificity	0.868 (0.855–0.880)	Beta	Schroy et al. (2013)
Large adenomas and CRC			
Sensitivity	0.950 (0.920–0.990)	Beta	Van Rijn et al. (2006)
Specificity	0.868 (0.855–0.880)	Beta	Schroy et al. (2013)
Increased rates after polypectomy (hazard ratio)			
Low risk	2 (1–3)	Log-normal	Assumed
High risk	3 (2–4)	Log-normal	Assumed
Costs ($)			
Colonoscopy	10,000 (9,000–11,000)	Log-normal	Assumed
Early clinical CRC, annual costs	21,524 (20,000–23,000)	Log-normal	Assumed
Late clinical CRC, annual costs	37,000 (35,000–39,000)	Log-normal	Assumed
Utilities			
Preclinical CRC	1.000 (0.980–1.000)	Log-normal	Assumed
Early clinical CRC	0.855 (0.700–0.900)	Log-normal	Ness et al. (1999)
Late clinical CRC	0.300 (0.200–0.400)	Log-normal	Ness et al. (1999)

### Uncertainty Quantification

We performed four different approaches to quantify the uncertainty of the two types of parameters—calibrated parameters and external (i.e., CEA) parameters. The first approach for uncertainty quantification considers uncertainty in both types of parameters, with uncertainty of the calibrated parameters characterized by their joint posterior distribution obtained from the IMIS algorithm. The second approach only considers uncertainty in the external parameters while fixing the calibrated parameters at the *maximum-a-posteriori* (MAP) estimate, defined as the parameter with the highest posterior density. The third approach considers uncertainty only in the calibrated parameters characterized by their joint posterior distribution and no uncertainty in the external parameters, fixed at their mean values. The fourth approach considers uncertainty in both types of parameters, but instead of using the IMIS posterior distribution of the calibrated parameters, we constructed distributions based solely on the IMIS posterior moments (i.e., means and standard deviations) and the type of calibrated parameters ignoring correlations.

We conducted a PSA to evaluate the impact of uncertainty in model parameters on the cost-effectiveness of 10-years colonoscopy screening vs. no screening for CRC. A separate PSA was performed for the four different approaches to quantify the uncertainty of the two types of parameters. We used EMEWS to distribute the samples of each PSA across HPC resources.

### Value of Information Analysis

We quantified the theoretical value of eliminating uncertainty in the external and calibrated model parameters using VOI analysis. VOI measures the losses (i.e., foregone benefits) from choosing a strategy given imperfect information (Raiffa and Schlaifer, 1961), providing the amount of resources a decision maker should be willing to spend to obtain information that would reduce the uncertainty. Specifically, we estimated the value of eliminating parametric uncertainty (i.e., the EVPI) in the cost-effectiveness of a 10-years colonoscopy screening strategy. This entailed computing the difference in net benefit between perfect information and current information (Oostenbrink et al., 2008). The EVPI was calculated across a wide range of willingness-to-pay (WTP) thresholds (Eckermann et al., 2010). We repeated this VOI analysis for the different approaches to characterize the uncertainty of the calibrated and external parameters.

## Results

**FIGURE 2:**
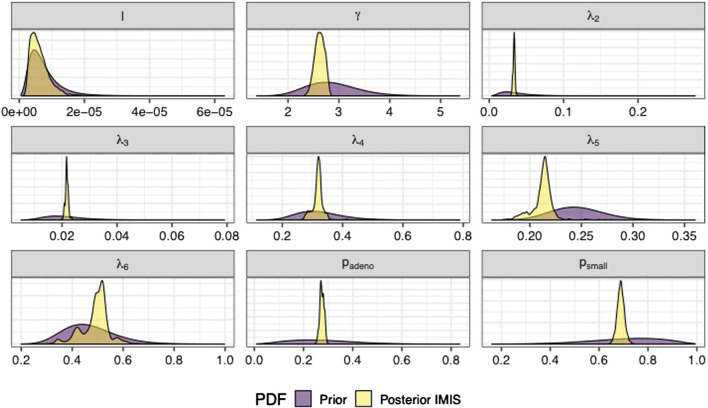
Prior and posterior marginal distributions of calibrated parameters of the microsimulation model of the natural history of CRC.

We sampled 5,000 parameter sets from the posterior distribution using IMIS, including 3,241 unique parameter sets with an expected sample size (ESS) of 2,098. With the sample from the posterior distribution, we estimated posterior means and standard deviations, MAP estimates, and 95% credible intervals (CrI) for all calibrated parameters (Table 3). The posterior means of the calibrated parameter were similar to the prior means (Table 3). Still, the major contrast is that the width of the posterior distributions shrunk, meaning that the calibration targets informed the calibrated parameters through a Bayesian updating (Figure 2).

**TABLE 3:** Posterior means, standard deviations, maximum-a-posteriori (MAP) estimate and 95% credible interval (CrI) of calibrated parameters of the microsimulation model of the natural history of CRC.

Parameter	Mean	SD	MAP	95% CrI	
				LB	UB
$p_{adeno}$	0.264	0.008	0.264	0.248	0.281
$p_{small}$	0.706	0.019	0.711	0.667	0.741
l	6.24E−06	3.16E−06	4.52E−06	1.92E−06	1.41E−05
γ	2.639	0.112	2.635	2.432	2.877
$\lambda_{2}$	0.035	0.002	0.035	0.031	0.039
$\lambda_{3}$	0.021	0.001	0.021	0.020	0.023
$\lambda_{4}$	0.374	0.036	0.368	0.310	0.448
$\lambda_{5}$	0.247	0.021	0.251	0.209	0.288
$\lambda_{6}$	0.457	0.076	0.435	0.345	0.664

**FIGURE 3:**
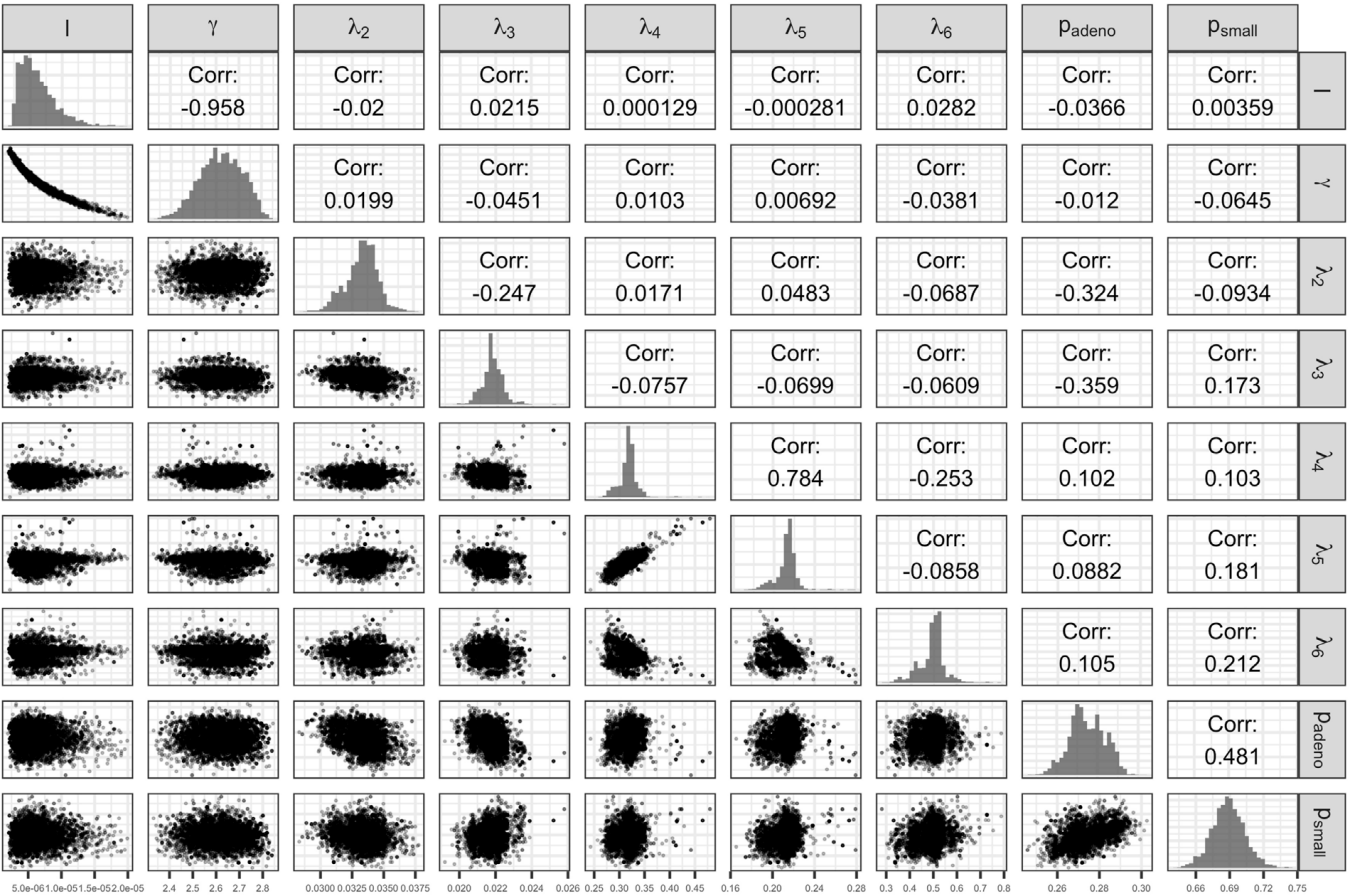
Scatter plot of pairs of deep model parameters with correlation coefficient and posterior marginal distributions.

The Bayesian calibration also correlated the parameters, showing the dependency among some of them (Figure 3). There are pairs of parameters with high correlation. The scale and shape parameters of the Weibull hazard function for the age of onset of adenomas, l , and γ , respectively, have the highest negative correlation of −0.958. The high correlation results from the calibration of the microsimulation model of the natural history of CRC being non-identifiable when calibrating all 9 parameters to all the targets. The transition rates from early preclinical CRC to late preclinical and early clinical have a correlation of 0.784. The prevalence of adenomas and the proportion of small adenomas at age 50, which inform the initial distribution of the cohort across the adenoma health states, also have a high correlation of 0.482. These high correlations result from the model calibration being non-identifiable. In a previous study, we found that the estimation of the 9 parameters of this model structure is non-identifiable via. calibration because the relationship between the parameters is highly colinear when using the current four calibration targets (Alarid-Escudero et al., 2018).

**FIGURE 4:**
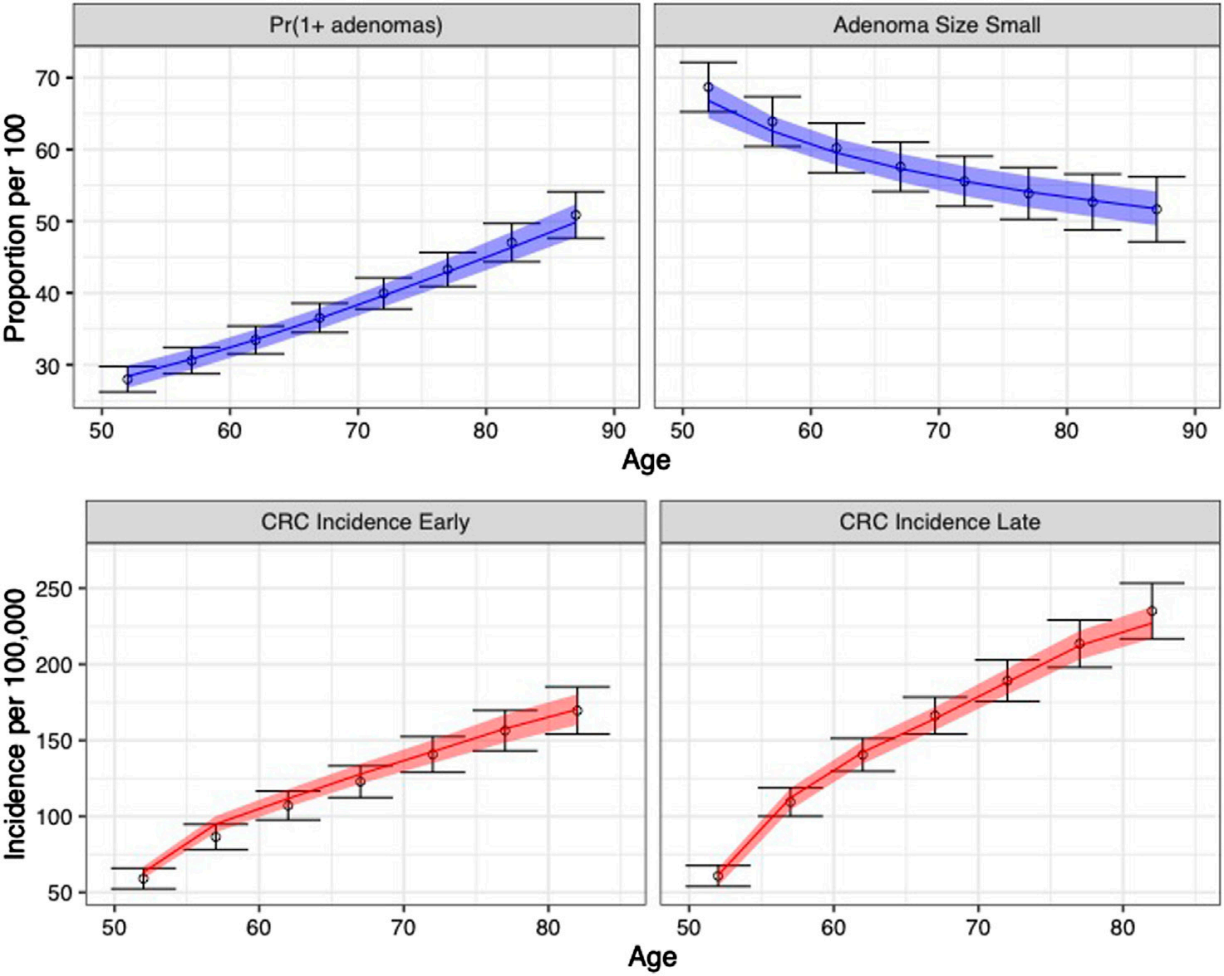
Comparison between posterior model-predicted outputs and calibration targets. Calibration targets with their 95% CI are shown in black. The shaded area shows the 95% posterior model-predictive interval of the outcomes and colored lines shows the posterior model-predicted mean based on 5,000 simulations using samples from the posterior distribution. Upper panel refers to adenoma-related targets and lower panel refers to CRC incidence targets by stage.

The calibrated model accurately predicted the calibration targets for both the means and the uncertainty intervals. Figure 4 shows the internal validation of the calibrated model by comparing calibration targets with their 95% confidence interval (CI) and the model-predicted posterior means together with their 95% posterior PI.

**FIGURE 5:**
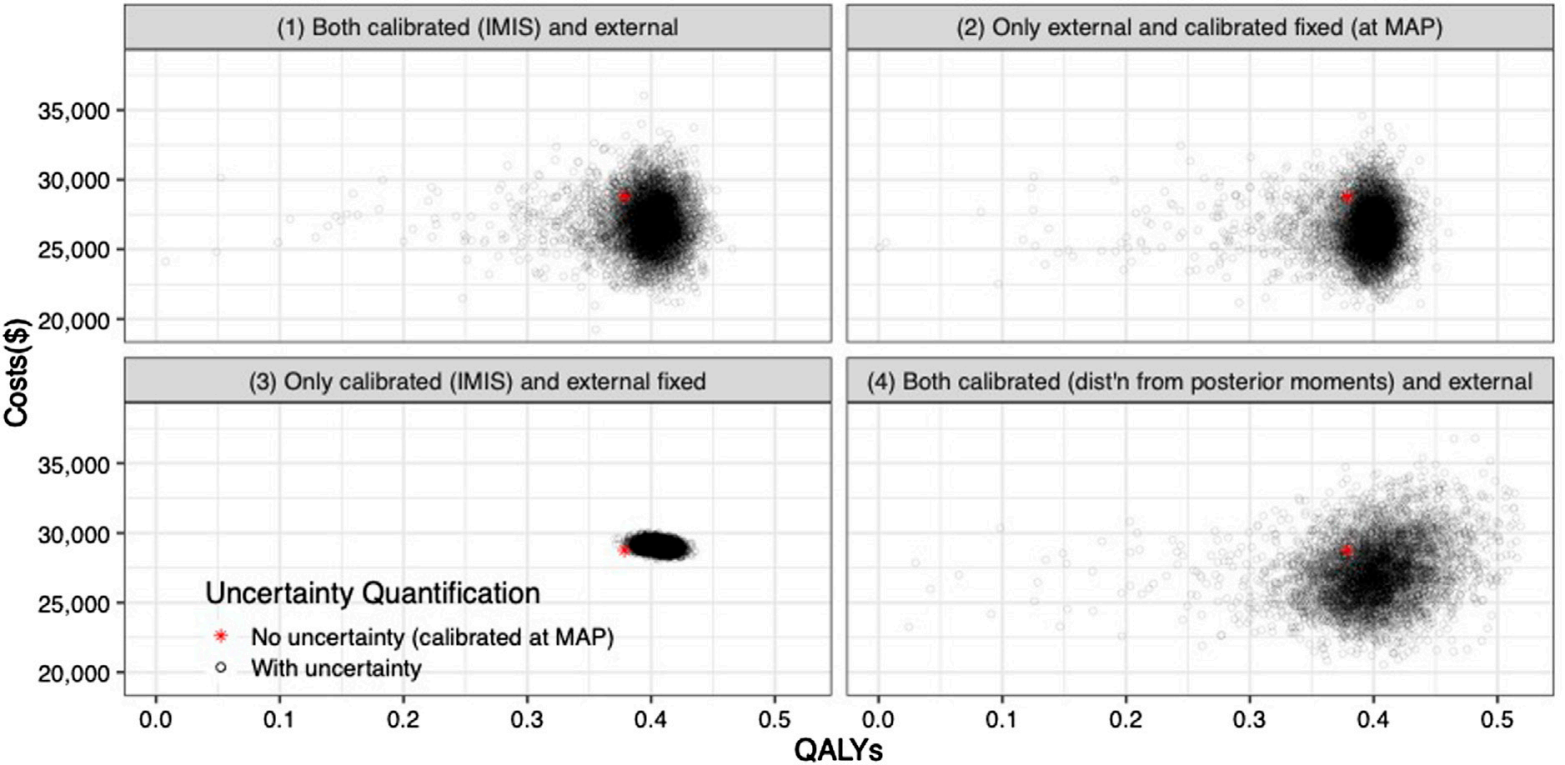
Incremental costs and incremental QALYs of 10-years colonoscopy screening vs. no screening under different assumptions of characterization of the uncertainty of both calibrated and external parameters. The red star corresponds to the incremental costs and incremental QALYs evaluated at the maximum-a-posteriori estimate of the calibrated parameters and the mean values of the external parameters.

The joint distribution of the incremental quality-adjusted life years (QALYs) and incremental costs of the 10 years colonoscopy screening strategy vs. the no-screening strategy resulting from the PSA for the four uncertainty quantification approaches of the calibrated parameters are shown in Figure 5. When accounting for the uncertainty on the external parameters, there is little difference in the spread of the CEA outcomes when considering the joint distribution of the calibrated parameters vs. using only the MAP estimates (approaches 1 and 2 on the top row of Figure 5, respectively). The joint distribution of the outcomes is slightly wider when considering uncertainty on all parameters compared to when fixing the calibrated parameters at their MAP estimate. The third approach reflects the impact of only varying the calibrated parameters on the joint distribution of incremental QALYS and incremental costs, which is much narrower than approaches 1 and 2. The fourth approach, which characterizes uncertainty of the calibrated parameters using the method of moments without accounting for correlation, has the widest spread on the distribution of the outcomes.

**FIGURE 6:**
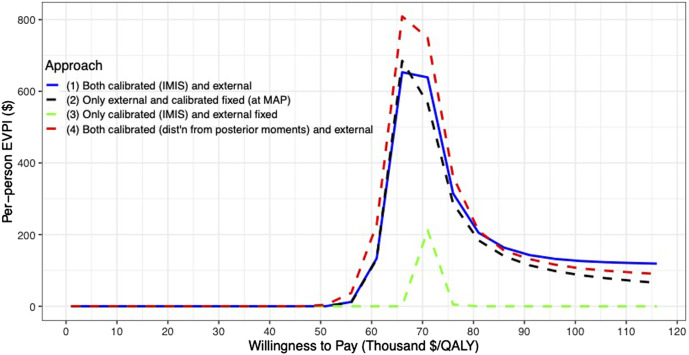
Per-patient EVPI of 10-year colonoscopy screening vs no screening under different approaches to characterize the uncertainty of both the calibrated and external parameters.

For the VOI analysis, we found value in eliminating uncertainty by having a positive EVPI in the parameters of the CEA of the 10-years colonoscopy screening strategy (Figure 6). However, the value varies by uncertainty quantification and WTP threshold. The first and second approaches to uncertainty quantification had similar EVPI, reaching their maximum of $653 and $685, respectively, at a $66,000/QALY WTP threshold. For WTP thresholds greater than $66,000/QALY, the first approach had a higher EVPI than the second approach. When we consider only the uncertainty for the calibrated parameters (approach 3), the EVPI is the lowest across all WTP thresholds with an EVPI of $0.1 at a WTP threshold of $66,000/QALY and reaching its highest of $212 at a WTP threshold of $71,000/QALY. The fourth approach reaches a maximum of $809 at a WTP threshold of $66,000/QALY and is the highest compared to the other approaches up to a WTP threshold of $81,000/QALY, at which the first approach has the highest EVPI.

## Discussion

In this study, we characterized the uncertainty of a realistic microsimulation model of the natural history of CRC by calibrating its parameters to different targets with varying degrees of uncertainty using a Bayesian approach on an HPC environment using EMEWS. We also quantified the value of the uncertainty of the calibrated parameters on the cost-effectiveness of a 10-year colonoscopy screening strategy with a VOI analysis. EMEWS has been previously used to calibrate other microsimulation DMs (Rutter et al., 2019; Rutter et al., 2019) but has not been previously used to conduct a PSA with the calibrated parameters and calculate the VOI. Although Bayesian calibration can be a computationally intensive task, we reduce the computation time by evaluating the likelihood of different parameter sets in multiple cores simultaneously on an HPC setup, which IMIS allows.

We found that different characterizations of the uncertainty of calibrated parameters affect the expected value of reducing uncertainty on the CEA. Ignoring inherent correlation among calibrated parameters on a PSA overestimates the value of uncertainty. When the full posterior distribution of the calibrated parameters is not readily available, the MAP could be considered the best parameter set. In our example, not considering the uncertainty of calibrated parameters on the PSA did not seem to have a meaningful impact on the uncertainty of the CEA outcomes and the EVPI of the screening strategy. The uncertainty associated with the natural history was less valuable than the uncertainty of the external parameters. However, these results should be taken with caution because this analysis is conducted on a fictitious model with simulated calibrated targets. Modelers should analyze the impact of a well-conducted characterization of the uncertainty of calibrated parameters on CEA outcomes and VOI measures on a case-by-case basis.

There are examples of calibrated parameters being included in a PSA. For instance, by taking a certain number of good-fitting parameter sets (Kim et al., 2007; Kim et al., 2009), bootstrapping with equal probability good-fitting parameter sets obtained through directed search algorithms (e.g., Nelder-Mead) (Taylor et al., 2012), or conducting a Bayesian calibration, which produces the joint posterior distribution of the calibrated parameters (Menzies et al., 2017). However, this is the first manuscript to conduct a PSA and VOI analysis using distributions of calibrated microsimulation DM parameters that accurately characterize their uncertainty.

Currently, Bayesian calibration of microsimulation DMs might not be feasible on regular desktops or laptops. To circumvent current computational limitations from using Bayesian methods in calibrating microsimulation models, surrogate models -often called metamodels or emulators-have been proposed (O’Hagan et al., 1999; O’Hagan, 2006; Oakley and Youngman, 2017). Surrogate models are statistical models like Gaussian processes (Sacks et al., 1989a; Sacks et al., 1989b; Oakley and O’Hagan, 2002) or neural networks (Hauser et al., 2012; Jalal et al., 2021) that aim to replace the relationship between inputs and outputs of the original microsimulation DM (Barton et al., 1992; Kleijnen, 2015), which, once fitted, are computationally more efficient to run than the microsimulation DM. Constructing an emulator might not be a straightforward task because the microsimulation DM still needs to be evaluated at different parameter sets, which could also be computationally expensive. Furthermore, the statistical routines to build the emulator may not be readily available in the programming language in which the microsimulation DM is coded. These are situations where EMEWS can be used to construct metamodels efficiently; however, this is a topic for further research.

Researchers might actively avoid questions that would require HPC due to the perceived difficulties involved or make do with less-than-ideal smaller-scale analyses (e.g., choosing the maximum likelihood estimate or a small set of parameters instead of the posterior distribution for uncertainty quantification) and the robustness of the conclusions can suffer as a result.

In this article, we showed that EMEWS could facilitate the use of HPC to implement computationally demanding Bayesian calibration routines to correctly characterize the uncertainty of the calibrated parameters of microsimulation DMs and propagate it in the evaluation of CEA of screening strategies and quantify their value of information. This study’s methodology and results could guide a similar VOI analysis on CEAs using microsimulation DMs to determine where more research is needed and guide research prioritization.

## Data Availability

The raw data supporting the conclusion of this article will be made available by the authors, without undue reservation.
